# Evaluation of Response to Neoadjuvant Chemoradiotherapy in Locally Advanced Rectal Cancer through Shear-wave Elastography

**DOI:** 10.2174/0115734056327323250108055841

**Published:** 2025-03-17

**Authors:** Qingfu Qian, Minling Zhuo, Yue Yu, Ensheng Xue, Xiaodong Lin, Zhikui Chen

**Affiliations:** 1 Department of Ultrasound, Fujian Medical University Union Hospital, Fuzhou, Fujian, China

**Keywords:** Endorectal ultrasound, Elastography, Rectal cancer, Neoadjuvant chemoradiotherapy, Regression, Complete response

## Abstract

**Background:**

There remains a lack of methods to accurately assess the efficacy of neoadjuvant chemoradiotherapy for locally advanced rectal cancer.

**Objective:**

This study aimed to investigate the value of shear-wave elastography in evaluating the treatment response to neoadjuvant chemoradiotherapy for locally advanced rectal cancer.

**Materials and Methods:**

This prospective observational study enrolled 275 patients with locally advanced rectal cancer who received neoadjuvant chemoradiotherapy during September 2021–March 2023. All patients underwent endorectal ultrasound and shear-wave elastography examination before total mesorectal excision. Clinical baseline data, endorectal ultrasound, and shear-wave elastography examination data were collected from all patients. The independent predictors of complete response were analyzed and screened, followed by the establishment of a logistic regression model. The diagnostic efficacy of the model was compared with that of radiologists.

**Results:**

The results of binary multivariate logistic regression suggested that the mean elastography value of the tumor lesion acted as an independent predictor for the diagnosis of complete response [OR: 0.894 (0.816, 0.981)]. The optimal cutoff value was 14.6 kPa. The area under the receiver operating characteristic curve of the model for predicting complete response in the training and test cohorts was 0.850 and 0.824, respectively. The diagnostic accuracy of the model was significantly higher than that of radiologists (P < 0.001).

**Conclusion:**

Shear-wave elastography can be used as a feasible method to evaluate the complete response of locally advanced rectal cancer after neoadjuvant chemoradiotherapy.

## 1 INTRODUCTION

Colorectal cancer is the third-most common malignant tumor in the world and is associated with the second-highest mortality rate [1]. Neoadjuvant Chemoradiotherapy (nCRT), followed by Total Mesorectal Excision (TME), has become the standard treatment for Locally Advanced Rectal Cancer (LARC) [2-4]. Following nCRT, varying degrees of relief and downstaging are usually achieved in rectal cancer, which then reduces the scope of surgery, increases the possibility of preserving the anal sphincter, reduces surgical complications, and improves patient prognosis.

After nCRT, an assessment of the response to treatment is usually required. Tumor Regression Grade (TRG) and TNM stage are two commonly used methods to evaluate the response to nCRT. The TRG system is primarily used to qualitatively evaluate the degree to which tumor cells are replaced by fibrosis [5], which reflects the efficacy of neoadjuvant therapy. Past studies have demonstrated that the TRG of rectal cancer is closely related to prognostic indicators, such as disease-free survival and overall survival [6, 7]. TRG 0 represents that the tumor has achieved a complete response after nCRT, signifying an optimal treatment response and an excellent survival outcome. High TRG usually indicates a higher risk of poor survival and disease progression, and patients may need to change their treatment course. It has been shown that tumor regression may not always be accompanied by T downstaging due to tumor fragmentation after nCRT [8]. Thus, even TRG 1 (near-complete response) does not always indicate a good prognosis [8, 9].

Patients with an apparent complete clinical response to nCRT are usually required to undergo radical resection. However, local excision has been found to be an acceptable curative treatment for carefully selected patients with cT0-1 that reduces the operative risk and functional sequelae resulting from total mesorectal excision [2]. Furthermore, some studies suggest that a “watch and wait” non-operative management approach may help avoid surgical complications in patients who achieve a complete response [10, 11]. Therefore, accurate preoperative assessment of the treatment response can provide information about the efficacy of treatment regimens and can provide a reference for the selection of subsequent treatment strategies for patients. However, reliable ways to accurately identify patients with a complete response continue to be lacking.

Some researchers found that Shear-wave Elastography (SWE) measurements of rectal lesions could help determine the T-downstaging of rectal cancer after nCRT [12], which displayed the potential of SWE to evaluate the response to nCRT. However, whether SWE can accurately assess TRG 0 of rectal cancer after nCRT has not yet been reported. This study aimed to investigate the value of SWE in evaluating the complete response to nCRT for LARC.

## 2 MATERIALS AND METHODS

### 2.1 Patients

This was a prospective observational study. The study was approved by the ethics committee of the investigator’s hospital. Informed consent was obtained from all participants.

This study recruited 496 consecutive patients with LARC who received nCRT at our hospital from September 2021 to March 2023. The study included patients (1) who had been pathologically diagnosed with rectal adenocarcinoma before nCRT; (2) in whom LARC was clinically assessed based on the results of comprehensive examinations, such as ERUS and pelvic MRI, before nCRT; (3) who were scheduled to undergo Total Mesorectal Excision (TME) after nCRT; (4) who underwent routine ERUS and SWE examinations within 2 weeks before surgery. Patients who (1) did not receive standard nCRT; (2) did not undergo TME; (3) simultaneously had other diseases that affected rectal cancer treatment; and (4) failed to undergo SWE examination (*e.g*., ultra-low rectal cancer, rectal stenosis, and rectal stent placement) were excluded from the study. In total, 275 consecutive participants with LARC were enrolled after screening in accordance with the inclusion and exclusion criteria. The patients were randomly stratified and sampled at a 3:2 ratio into the training and test cohorts with 166 and 109 patients, respectively. Fig. (**1**) presents the detailed patient recruitment process.

### 2.2 Routine ERUS and SWE Examination

All patients underwent routine ERUS and SWE examinations 8 weeks after the completion of nCRT. These examinations were conducted by two radiologists blinded to the pathological findings. Both the radiologists had more than 10 years of experience in ERUS diagnosis.

To ensure the image quality, all patients were subjected to a cleansing enema before the examination, and 200 mL of warm water was introduced through the anus to establish an acoustic window in the rectal cavity. The HITACHI Preirus Ultrasound Diagnostic Device (Hitachi Medical, Tokyo, Japan), equipped with an intracavitary ring probe (R54AW-19; frequency: 5–10 MHz) and a head scanning probe (EUP-V53W; frequency: 4–8 MHz), was employed in the routine ERUS examination. The routine ultrasound features of the rectal cancer lesion after nCRT were evaluated and recorded, which also included the location of the tumor lesion (the distance between the lower edge of the lesion and the anal margin of ≤5 cm was defined as the low position, 5.1–10 cm as the middle position, and 10.1–15 cm as the upper position), lesion length, thickness, and suspicious metastatic lymph nodes (round or elliptical hypoechoic nodes with a long axis distance of >0.5 cm and no lymphatic portal structure visible [13]). The criteria for ERUS evaluation of the complete response were the absence of evidence of rectal cancer on ultrasound imaging.

After the routine ERUS examination, an SWE examination was performed using the SuperSonic Aixplorer Ultrasound Diagnostic Device (SuperSonic Imagine, Aix-en-Provence, France) equipped with an intracavitary probe (SE12-3; frequency: 3–12 MHz). The section with the deepest tumor infiltration depth on the image was selected and then converted to the SWE examination mode. The probe was then placed close to the lesion to avoid artifact production, but no pressure was applied. The maximum SWE range was uniformly adjusted to 100 kPa, and the sampling box size was adjusted to cover the tumor lesion and the surrounding mesenteric tissues. After the color signal of the image was stabilized, three dynamic videos (each of 5–10 s) were stored. In addition, three dynamic videos of the distant rectal wall and mesenteric tissue at 2 cm from the tumor were stored. After the examination, the SWE-examining radiologist re-reviewed the stored dynamic videos and selected three stable images with good color filling from each dynamic video for measuring the SWE parameters. Ultimately, nine SWE images containing the tumor lesion and peritumoral mesangial tissues and nine SWE images containing the distant rectal wall and distant mesangial tissues were obtained for each patient.

The Region of Interest (ROI) for the tumor lesion was outlined using the “Q-box trace” quantitative tool provided by the equipment, and the ROI was enclosed around the entire tumor lesion. The “Q-box” quantification tool was used to measure the SWE parameters of the distant rectal wall, and the circular ROI covered the entire rectal wall. The other measurement sites, including the mesenteric tissues surrounding the lesion within 5 mm and distant mesangial tissues, were measured by the “Q-box” measurement tool, while the ROI diameter was uniformly set to 3 mm. In this study, the mean elastography value (Emean) was used as the study variable, and the median of these SWE parameters was recorded in kPa. Moreover, the ratios of the elastic values between the tumor lesion and distant rectal wall tissues (ERTD) and those between the peritumoral mesenteric tissue and distant mesenteric tissue (ERPD) were calculated.

### 2.3 Evaluation of TRG 0 by ERUS

All ERUS images in the test cohort were independently evaluated by radiologists. The criteria for evaluating Tumor Regression Grade (TRG) 0 were defined as the absence of any evidence of rectal cancer on ERUS.

### 2.4 Collection of Clinical Baseline Data

The clinical baseline data, including patients' gender, age, and preoperative serum Carcinoembryonic Antigen (CEA) level, were collected by reviewing medical records and laboratory test results.

### 2.5 Analysis of the Repeatability of SWE Measurements

The repeatability of SWE measurements was evaluated using the Intraclass Correlation Coefficient (ICC). Four weeks after the first measurement, the same radiologist who performed the SWE examination reviewed the stored videos on the equipment, re-selected the images, and re-measured the SWE values. The intraobserver ICC was calculated for the two measurements. Another ERUS radiologist repeated these steps, and the interobserver ICC was calculated for two SWE measurements. ICC ≥0.75 indicated a high consistency, 0.5–0.74 indicated moderate consistency, and ≤0.5 indicated low consistency.

### 2.6 Pathological Assessment of Response to Neoadjuvant Therapy

The TRG was assessed using the criteria proposed by the American Joint Committee on Cancer [14] (TRG 0: complete response, no viable cancer cells; TRG 1: near-complete response, only single cancer cells or rare small cell clusters remaining; TRG 2: partial response, residual cancer with marked tumor regression, and not just single cancer cells or rare small cell clusters; and TRG 3: poor or no response, no marked tumor regression, and a large amount of residual cancer tissues). Patients were classified as TRG 0 or non-TRG 0 groups based on whether they showed a complete response.

### 2.7 Model Establishment and Validation

Single-factor analysis and binary multivariate logistic regression analysis were performed for screening the collected clinical baseline data (gender, age, and preoperative serum CEA level) and ERUS data (tumor location, length, thickness, blood flow signal, and suspected metastatic lymph nodes) as well as SWE measurements. A logistic regression model was established according to the selected features. During the model training process, 5-fold cross-validation and grid search were performed to identify the optimal hyperparameters. Independent test data were employed to evaluate the model’s performance.

### 2.8 Statistical Analysis

PASS 15.0 software was used to calculate the sample size in this study. It was arbitrarily assumed that the AUC of the model would be at least 0.85, with a prespecified power of 0.80 and α = 0.05. A two-sided z-test was employed for analysis. The result of our calculations indicated that to detect a difference of 0.10, at least 194 cases (39 participants with TRG 0 and 154 participants with non-TRG 0) were needed to determine whether TRG 0 was achieved after neoadjuvant chemoradiotherapy in rectal cancer.

R software (version 4.3.1; http://www.r-project.org/) was used for conducting all statistical analyses. Continuous variables were expressed as mean ±SD (normally distributed) or the median and quartile range (non-normally distributed), with group comparisons being performed using the Student’s *t*-test or Mann–Whitney U-test. Categorical variables were expressed as the frequency or percentage, with group comparisons being performed using the Chi-square test. Receiver Operating Characteristic (ROC) curves were plotted for various SWE parameters. The Area Under the Curve (AUC) and 95% confidence interval of AUC were calculated. The optimal cutoff value for predicting TRG 0 was calculated using the maximum Youden's index. The accuracy, sensitivity, specificity, positive predictive value, and negative predictive value were used to evaluate the diagnostic performance of the model and the radiologists in the test cohort. The diagnostic accuracies of the model and radiologists were compared using the paired Chi-square test. A two-tailed *P* < 0.05 was considered to indicate statistical significance.

## 3 RESULTS

### 3.1 Baseline Clinical Data

In total, 275 patients with LARC (average age: 60 years, age range: 28–81 years) were finally enrolled in this study. After nCRT, the TRG 0 and non-TRG 0 groups accounted for 26.5% (73/275) and 73.5% (202/275) of the total patients, respectively. Table **1** summarizes the baseline clinical characteristics of all patients, and no statistically significant differences were observed in the distribution of clinical, ERUS, and pathological features between the training and test cohort patients (*P* > 0.05).

### 3.2 Intraobserver and Interobserver Repeatability Analyses of SWE Measurements

As shown in Table **2**, the SWE parameters measured by the same radiologist in two measurements (intraobserver repeatability) and those measured by two radiologists (interobserver repeatability) were highly consistent.

### 3.3 Univariate Analysis and Multivariate Analysis

Univariate analysis was performed on the clinical baseline data, ERUS examination data, and SWE measurements of the patients in the training cohort. As shown in Table **3**, tumor location, length, thickness, and suspicious metastatic lymph nodes exhibited statistically significant differences between the two groups (*P* < 0.05). All SWE parameters in the TRG 0 group were significantly lower than those in the non-TRG 0 group.

All significant features were included in the binary multivariate logistic regression analysis. Tumor lesion Emean [OR: 0.894 (0.816, 0.981), *P* = 0.018] acted as an independent predictor of TRG 0.

### 3.4 Model Establishment and Validation

**Fig. (2):**
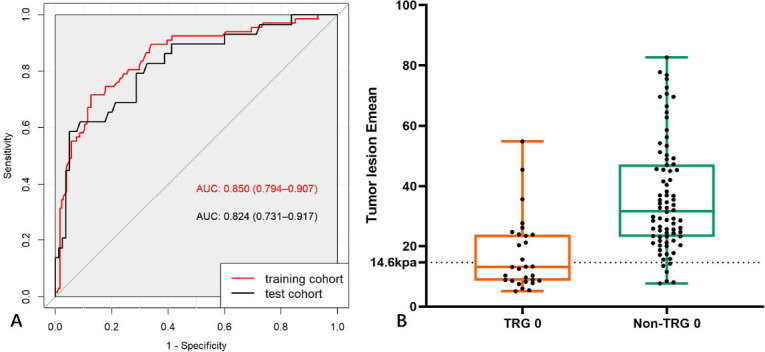
**A**). ROC curves in the training and test cohorts. **B**). Box diagram distribution of the SWE value of different pathological indicators in the test cohort.

**Fig. (3):**
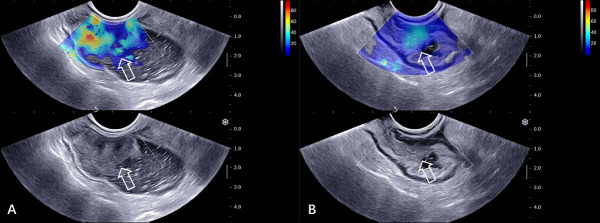
Examples of radiologists predicting TRG 0 incorrectly but model correctly. **A**), A 34-year-old female participant was evaluated as locally advanced rectal cancer before nCRT, whose tumor lesion Emean was 36.1 kPa. **B**), After nCRT, the tumor lesion was evaluated as TRG 0 by the radiologist. However, the tumor lesion Emean detected by SWE was 32.4kPa, and the model suggested that it was non-TRG 0.

Based on the results of the binary multivariate logistic regression analysis, a logistic regression model was established using tumor lesion Emean. The optimal cutoff value of tumor lesion Emean was 14.6 kPa. The AUC of the model for predicting TRG 0 of rectal lesions in the training and test cohort was 0.850 and 0.824, with an accuracy of 0.830 and 0.835, respectively (Figs. **2** and **3**, Table **4**).

### 3.5 Evaluation of Response to nCRT for Rectal Cancer by Radiologists

In the test set, radiologists correctly evaluated TRG 0 in 78 cases, overestimated 16, and underestimated 15. There were significant differences in the classification performance between radiologists and the model in predicting whether TRG 0 was achieved after neoadjuvant therapy for LARC (the diagnostic accuracy was compared, *P* < 0.001) (Tables **4** and **5**).

## 4 DISCUSSION

In this study, we prospectively enrolled 275 patients with LARC who underwent nCRT. Their clinical data, conventional ERUS, and SWE data were collected and analyzed to screen for features predicting TRG 0. According to these results, tumor lesion Emean could be used as an independent predictor of complete response to nCRT for rectal cancer, and the developed logistic regression model exhibited good predictive performance.

Several studies have indicated a close relationship between preoperative serum tumor marker levels and the prognosis of rectal cancer [15-17]. Specifically, elevated serum CEA levels in rectal cancer patients after nCRT have been found to be associated with a high TRG [18]. However, unlike previous studies, the results of univariate analysis in this study showed that preoperative CEA levels did not significantly aid in determining whether a patient achieved TRG 0 after nCRT.

ERUS offers the advantage of high resolution and can display the hierarchical structure of the rectal wall, which is one of the important methods for the preoperative evaluation of rectal cancer. However, rectal tumors often undergo a series of changes, such as tumor necrosis, fibrosis, and inflammation of the rectal wall and peritumoral mesenteric tissues after nCRT. The ultrasound images of residual tumor tissues, fibrotic lesions, and inflammatory tissues can all be seen as hypoechoic tissues. Therefore, it is often difficult to distinguish cancer tissues from other tissues by using the conventional ERUS examination, which limits the value of ERUS in evaluating the efficacy of nCRT in rectal cancer. Despite the extensive diagnostic experience of the participating radiologists in this study, the accuracy, sensitivity, and positive predictive value for the diagnosis of TRG0 remain insufficient.

The conventional ERUS features gathered in this study detected significant differences in tumor lesion location, length, thickness, blood flow signal, and suspicious metastatic lymph nodes between the TRG 0 and non-TRG 0 groups based on the univariate analysis results. However, these conventional ERUS features were all excluded as confounding factors in the multivariate regression analysis. Ultimately, only the SWE index was screened out as an independent predictor of TRG 0 in the multivariate regression analysis. This statistical result illustrates the limitation of conventional ERUS features in evaluating the efficacy of nCRT for rectal cancer. We finally selected the tumor lesion Emean as the variable in the prediction model.

Unlike ERUS, SWE evaluates the stiffness of the tissues by measuring Young's modulus value of the ROI, which can indirectly reflect the treatment response of the tumor. The use of SWE for assessing the effectiveness of neoadjuvant therapy for breast cancer has achieved numerous research outcomes. For example, SWE examination can help predict the efficacy of neoadjuvant therapy, evaluate axillary lymph nodes, and predict prognosis [15-23]. Meanwhile, some studies have demonstrated that, after neoadjuvant therapy, the elastic values of breast cancer lesions with different residual cells are different, thereby exhibiting the potential to predict TRG through SWE [24, 25]. Our study confirmed the value of SWE in evaluating the response to nCRT in rectal cancer. The tumor lesion Emean-based model constructed in this study could help determine whether the tumor lesion achieved the complete response. The AUC and ACC for predicting TRG 0 were 0.824 and 0.835, respectively.

Although the accuracy of our model in diagnosing TRG 0 was higher than that of radiologists (P < 0.05), some patients could not be accurately evaluated in the test cohort, considering that 12 TRG 0 lesions were overestimated by the model as non-TRG 0, which yielded a lower diagnostic sensitivity. Among them, 8 patients still had edema in the lesions, and the remaining 4 lesions showed calcification in the pathology, which resulted in high Emean values of the tumor lesions. In addition, 6 non-TRG 0 lesions were misclassified as TRG0, as the postoperative pathology showed that residual cancer cells accounted for 1% to 2% of the lesion. When a small number of residual cancer cells are located in the tumor lesion without any obvious fibrous tissue hyperplasia, inflammatory edema, and calcification, the SWE measurement may be similar to that of the complete remission lesions, resulting in the underestimation of the actual TRG.

Our study has involved some limitations. First, all cases in this study were sourced from a single center, which may have led to some bias in the selection of cases. Second, there was no additional external validation cohort data to further validate the predictive performance of the model in this study. Finally, the distribution was not balanced among the diverse grades; therefore, no further predictions could be made at the individual TRG level.

## CONCLUSION

In this study, the tumor lesion Emean-based model demonstrated a good prediction performance for the complete response of rectal cancer after nCRT, which can provide a reference for the subsequent treatment plan.

## Figures and Tables

**Fig. (1):**
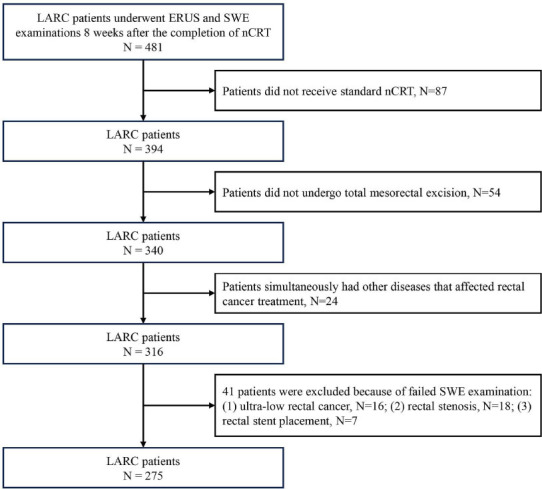
Flowchart depicting the patient recruitment process.

**Table 1:** The baseline characteristics of the study patients.

**Characteristics**		**Total** **(n=275)**	**Training Cohort** **(n=166)**	**Test Cohort** **(n=109)**	**P-value^†^**
Patient demographics					
Age (years, x±s) *	-	60.42± 10.48	60.58 ± 10.52	60.18 ±10.95	0.724
Sex	Male	175 (63.6)	105 (63.3)	70 (64.2)	0.870
-	Female	100 (36.4)	61 (36.7)	39 (35.8)	-
ERUS features					
Distances from the anal margin, n (%)	≤5.0cm	109 (39.6)	66 (39.8)	43 (39.4)	0.959
	5.1-15.0 cm	166 (60.4)	100 (60.2)	66 (60.6)	-
Length (cm, x±s)*	-	2.70 ±0.99	2.73±0.99	2.64±1.00	0.422
Thickness (cm, x±s)*	-	1.07±0.37	1.07 ±0.34	1.06±0.41	0.801
Blood flow signal	Poor	40 (14.5)	19 (11.4)	21 (19.3)	0.072
-	Abundant	235 (85.5)	147 (88.6)	88 (80.7)	-
Suspected metastatic lymph node	Present	37 (13.5)	23 (13.9)	16 (14.8)	0.856
	Absent	238 (86.5)	143 (86.1)	95 (87.2)	-
Laboratory parameters					
CEA	<5	220 (80.0)	134 (80.7)	86 (78.9)	0.759
-	≥5	55 (20.0)	32 (19.3)	23 (21.1)	-
Histologic characteristics					
TRG	0	73 (26.5)	44 (26.5)	29 (26.6)	0.985
-	1-3	202 (73.5)	122 (73.5)	80 (73.4)	-

**Note:** *Data are expressed as mean ±standard deviation; the remaining data indicate numbers of patients, with percentages in parentheses. nCRT = neoadjuvant chemoradiotherapy, TRG = tumor regression grade, CEA = carcinoembryonic antigen.

**Table 2:** Analysis of intra-observer and inter-observer consistencies in SWE measurements.

**Groups**	**Tumor Lesion**	**Peritumoral Mesangial Tissues**	**Distant Rectal Wall**	**Distant Mesangial Tissue**
Intra-operator reliability	0.898 (0.860~0.916)	0.901 (0.840~0.938)	0.925 (0.860~0.952)	0.909 (0.822~0.945)
Inter-operator reliability	0.845 (0.763~0.852)	0.892 (0.832~0.935)	0.905 (0.833~0.947)	0.898 (0.807~0.939)

**Note**: Data in brackets are presented as 95% confidence intervals.

**Table 3:** Comparison of clinical, ERUS, and SWE examination data between the TRG 0 group and the non-TRG 0 group in the training set.

**Variables**	**TRG 0 (n=44)**	**Non-TRG 0 (n=122)**	***χ* ^*2*/*t/Z*^ value**	**P-value**
Age (years, x±s)*	60.23± 11.14	60.70± 10.34	0.257	0.797
Sex	-	-	3.105	0.078
Male	23	82	-	-
Female	21	40	-	-
CEA	-	-	2.409	0.121
≤5	39(88.6)	95 (77.9)	-	-
>5	5(11.4)	27(22.1)	-	-
Location of the tumor lesion	-	-	9.342	0.002
Low	26	40	-	-
Middle, upper	18	82	-	-
Length (cm, x±s)*	2.30±0.91	2.89±0.98	3.485	0.001
Thickness (cm, x±s)*	0.92±0.24	1.13±0.35	3.762	0.001
Blood flow signal	-	-	5.059	0.024
Poor	9	10	-	-
Abundant	34	112	-	-
Suspected metastatic lymph node	-	-	9.629	0.002
Present	0	23	-	-
Absent	44	99	-	-
Tumor lesion Emean†	8.15(5.58,14.38)	32.35(20.78,47.93)	-7.575	<0.001
Peritumoral mesenteric tissue Emean†	6.05(4.05,8.88)	30.50(7.98,55.5)	-5.768	<0.001
ERTD†	1.48(0.92,2.91)	5.95(3.50,9.05)	-7.372	<0.001
ERPD†	1.27(0.96,2.01)	6.43(1.74,11.74)	-5.543	<0.001

**Note:** * The Student’s *t*-test was used for comparison between the two groups. †The Mann–Whitney U-test was used for comparison between the two groups. ERTD = Emean ratio for the tumor versus the distant rectal wall; ERPD = Emean ratio for the peritumoral mesangial tissue versus the distant mesangial tissues.

**Table 4:** Comparison of the diagnostic performance of radiologist and model to predict TRG 0 in the test cohort.

	**Accuracy**	**Sensitivity**	**Specificity**	**PPV**	**NPV**
Radiologist	0.716	0.448	0.813	0.464	0.802
Model	0.835	0.586	0.925	0.739	0.860

Note: PPV = positive predictive value; NPV = negative predictive value.

**Table 5:** Comparison of the ability of radiologist and model to predict TRG 0 in the test cohort.

	**Radiologist**			**Model**		
**Pathology**	**TRG 0**	**Non-TRG 0**	**Total**	**TRG 0**	**Non-TRG 0**	**Total**
TRG 0	13	16	29	17	12	29
Non-TRG 0	15	65	80	6	74	80
Total	28	81	109	23	86	109

## Data Availability

The data that support the findings of this study will be available from the corresponding author [Z.C.] upon special request.

## LIST OF ABBREVIATIONS

AUC: Area under the receiver operating characteristic curve
CEA: Carcinoembryonic antigen
ERUS: Endorectal ultrasound
LARC: Locally advanced rectal cancer
nCRT: Neoadjuvant chemoradiotherapy
ROI: Region of interest
TRG: Tumor regression grade
SWE: Shear-wave elastography

## STANDARDS OF REPORTING

STROBE guidelines were followed.
